# The Development of a Vaccine Against Meningococcus B Using Reverse Vaccinology

**DOI:** 10.3389/fimmu.2019.00751

**Published:** 2019-04-16

**Authors:** Vega Masignani, Mariagrazia Pizza, E. Richard Moxon

**Affiliations:** ^1^GSK Vaccines, Siena, Italy; ^2^Department of Pediatrics, Oxford University, Oxford, United Kingdom

**Keywords:** 4CMenB vaccine, reverse vaccinology, strain coverage, cross protection, antigenic variability, *Neisseria meningitidis* serogroup B (MenB)

## Abstract

The discovery of vaccine antigens through whole genome sequencing (WGS) contrasts with the classical hypothesis-driven laboratory-based analysis of microbes to identify components to elicit protective immunity. This radical change in scientific direction and action in vaccine research is captured in the term *reverse vaccinology*. The complete genome sequence of an isolate of *Neisseria meningitidis* serogroup B (MenB) was systematically analyzed to identify proteins predicted to be secreted or exported to the outer membrane. This identified hundreds of genes coding for potential surface-exposed antigens. These were amplified, cloned in expression vectors and used to immunize mice. Antisera against 350 recombinant antigens were obtained and analyzed in a panel of immunological assays from which 28 were selected as potentially protective based on the -antibody dependent, complement mediated- serum bactericidal activity assay. Testing of these candidate vaccine antigens, using a large globally representative strain collection of Neisseria species isolated from cases of disease and carriage, indicated that no single component would be sufficient to induce broad coverage and that a “universal” vaccine should contain multiple antigens. The final choice of antigens to be included was based on cross-protective ability, assayed by serum bactericidal activity and maximum coverage of the extensive antigenic variability of MenB strains. The resulting multivalent vaccine formulation selected consisted of three recombinant antigens (Neisserial Heparin Binding Antigen or NHBA, Factor H binding protein or fHbp and Neisseria Adhesin A or NadA). To improve immunogenicity and potential strain coverage, an outer membrane vesicle component obtained from the epidemic New Zealand strain (OMVNz) was added to the formulation to create a four component vaccine, called 4CMenB. A series of phase 2 and 3 clinical trials were conducted to evaluate safety and tolerability and to estimate the vaccine effectiveness of human immune responses at different ages and how these were affected by various factors including concomitant vaccine use and lot-to-lot consistency. 4CMenB was approved in Europe in 2013 and introduced in the National Immunization Program in the UK starting from September 2015 when the vaccine was offered to all newborns using a 2, 4, and 12 months schedule., The effectiveness against invasive MenB disease measured at 11 months after the study start and 5 months after the second vaccination was 83% and there have been no safety concerns.

## Meningococcus B: The Last Frontier

The development of a meningococcal vaccine to protect against invasive disease caused by serogroup B strains of *Neisseria meningitidis* (MenB) represents a milestone in vaccinology. MenB is a major cause of sepsis and meningitis in North and South America, Canada, Europe, Australasia, and many other countries, but developing an effective vaccine was for many years an unsolved problem. The stumbling block was that, in contrast to all other variant capsular polysaccharides of the meningococcus for which effective conjugate vaccines were developed and licensed, the B polysaccharide does not induce an effective antibody response. A study of 50 healthy adults immunized with MenB polysaccharide showed that all but three of them failed to produce any antibodies (1) and even conjugation to tetanus toxoid failed to improve its immunogenicity (2). In contrast, in the 1960s, it was shown that adult military recruits immunized with plain MenC polysaccharide responded with copious amounts of antibody that protected against meningitis and sepsis (3). Further, a trial in Finland using unconjugated MenA polysaccharide also showed strong, protective immune responses even in young children (4). Why was the MenB such a poor immunogen? Scientists from Finland showed that the MenB capsular polysaccharide was identical to sugars found on the surface of many human cells especially, but not exclusively, in the brain during its pre-natal development (5, 6). It was concluded that inducing antibodies to MenB capsular polysaccharide ran the risk of damaging structures found on the surface of human cells and the authors proposed that the immune system had evolved tolerance to the B polysaccharide as a mechanism to avoid autoimmune pathology because of mimicry between components on the cell surface of human cells and surface structures of bacteria. The B polysaccharide is a homo-polymer of α (2 → 8) N-acetyl neuraminic acid, known also as polysialic acid (PSA), located on the surface of human cells. PSA has unusual and important biological properties. To allow intimate intercellular interactions, water must be excluded and PSA, richly hydrated, modulates the cell to cell signaling. Using antibodies or an enzyme that specifically destroys PSA, animal experiments have shown its key role in programming CNS development, including the migration of nerve cells, the connectivity between dendritic cells and the formation of junctions between muscle and nerves (7, 8).

Many scientists concluded that inducing antibodies to PSA in humans represented an unacceptable safety risk. A particularly alarming concern that consolidated opposition to using the polysaccharide as a vaccine concerned the risk to immunized women who become pregnant. Antibodies cross the placenta and reach the developing embryo, so antibodies resulting from immunization with B polysaccharide could disrupt CNS development in the unborn child, especially since the amount of PSA on neural tissues is known to be at its highest level during fetal development. But not all scientists were convinced that the evidence precluded using the B polysaccharide as a vaccine. Harold Jennings, one of the first to demonstrate that the B polysaccharide was inert as an immunogen had the idea of modifying the B polysaccharide by introducing an N-propionyl side chain with the aim of increasing immunogenicity and precluding cross reactivity to human cells (9). This formulation elicited functional antibody responses in mice, but not in humans (10).

John Robbins was strongly supportive of this approach and even today remains steadfast in his opinion that using the B polysaccharide as a vaccine would not be harmful. He and his collaborators have documented that humans, through exposure to naturally occurring antigens, make antibodies to the polysaccharide, yet do not have an increased susceptibility to auto-immune disease (11). But, for the majority of scientists, an alternative approach to a vaccine, one that avoided the use of the potentially harmful B polysaccharide, was considered imperative. Crucially, vaccine manufacturers were strongly influenced by safety concerns and were unwilling to embark on investing millions into a research and development programs that risked being derailed when ethical approval was sought to carry out the mandatory clinical trials in humans.

Over many years, research on alternative approaches to develop a vaccine that protected against invasive diseases caused by strains of MenB was undertaken. Efforts were largely directed to the non-capsular antigens, proteins, or lipopolysaccharide (in meningococcus, often referred to as lipo-oligosaccharide or LOS). This change brought about a radical conceptual shift; referring to these non-capsule based vaccines as “group B” vaccines is a misnomer as they do not contain the defining feature of MenB bacteria (the polysaccharide capsule), and the vaccines will also potentially protect against other capsular groups (A, C, W, and Y strains).

The ability of the antibodies induced by each vaccine antigen to activate complement and induce bactericidal activity, measured in the serum bactericidal assay (SBA), was shown to be predictive of protection in humans (12, 13). However, although the SBA was well-established in the context of conjugate vaccines, its credentials as a correlate of protection in the context of protein antigens required further, independent validation. This came from the evidence of Goldschneider (12, 13) and experience with outer membrane vesicles (OMVs) [in particular those used in Norway and New Zealand (14, 15)], whose protective efficacy was shown to be largely mediated by bactericidal antibodies to the meningococcal surface protein, PorA. Although a number of surface-exposed candidate antigens were identified (16–18), none possessed sufficient capacity to elicit cross-reactive bactericidal activity against the diversity of meningococcal strains, the *sine qua non* that was essential for developing an effective vaccine. However, OMVs, treated with detergents to extract LOS and decrease endotoxin activity, were safe and effective in preventing group B meningococcal disease (19–22); A variety of “tailor-made” MenB OMV vaccines have been developed and licensed to control epidemics dominated by a single clone. OMV vaccines have been used in Norway (23), Cuba (24), Chile (25), and New Zealand (26). MeNZB, which was implemented in New Zealand, was associated with substantial reductions in invasive meningococcal disease caused by an outbreak clone that reached an incidence of 17.4/100,000 of the population overall and more than 200/100,000 among some indigenous populations (27). In published studies, efficacy of two doses given to children 4 years or older, or to young adults, ranged from 57 to 83% but with the limitation that the protective, bactericidal responses of infants were specific for the major outer membrane porin protein (PorA) (14). Thus, the utility of OMV vaccines is limited to control clonal epidemics where disease is caused by strains expressing a PorA serosubtype matching that in the OMV vaccine. In an effort to broaden protection, OMV vaccines were prepared from several strains expressing distinct alleles of PorA, but the manufacturers of this multivalent formulation were not able to overcome a number of problems that included variable immunogenicity and consistency of formulation (28).

New technology was needed to overcome the impasse and this came about in 1995 when a team from The Institute for Genomic Research (TIGR) sequenced the complete genome of the human commensal-pathogen bacterium *Haemophilus influenzae* (29). The first completely assembled genome of a free-living organism was a revolution in biology; whole genome sequencing (WGS) transformed the scientific basis of epidemiology, diagnosis, and prevention of all diseases, including those caused by microbes. In the field of infectious diseases, WGS introduced a cost-effective method to acquire comprehensive information on pathogens and commensals, including those whose biology was elusive because they could not be cultivated in the laboratory. The implications of completing the first WGS were immediately apparent. The idea of using a genomics platform as a discovery tool to identify vaccine antigens was first explicitly published in 1997 (30) and the public health imperative to develop a vaccine to prevent invasive infections caused by MenB provided an ideal opportunity to exploit this concept. Scientists from Oxford University provided Venter and his team at The Institute for Genomics Research (TIGR) with DNA from a MenB strain (MC58) isolated from a UK outbreak of meningococcal disease (31). Preliminary genome sequence data, initially a modest 2-fold genome coverage, validated the potential of the approach by identifying a novel antigen (17, 18). Meantime, Italian scientists from Sclavo, Siena (led by Rino Rappuoli) had for several years dedicated their research efforts toward the development of meningococcal vaccines. In 1998, a collaboration between Chiron Vaccines (who acquired Sclavo), TIGR and Oxford University, carried out a comprehensive evaluation of all potential meningococcal vaccine antigens in the MC58 MenB strain, as described below. The WGS approach, highly sensitive but lacking the specificity to identify and prioritize antigens with respect to protective potential, was published in 2000 (32, 33). Antigen discovery through WGS contrasted with the hypothesis-driven classical laboratory based, bottom-up analysis of microbes to identify components that could elicit protective immunity. This radical change in scientific direction and action in vaccine research was subsequently captured in the term *reverse vaccinology* (34). Many of these were outer membrane proteins that had relatively low levels of surface expression, one reason why they had not been discovered before the use of WGS.

## From Bioinformatics to Biology

**Figure 1:**
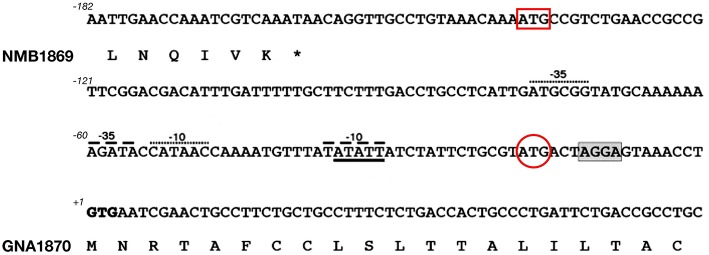
Challenges in annotating correct start codons: the case of GNA1870. Nucleotide sequence of the intergenic region between nmb1869 and *gna1870* genes in MenB strain MC58. The DNA sequence and deduced amino acid translation of the C-terminus of NMB1869 and N-terminal predicted lipoprotein signal peptide of GNA1870 are shown. The predicted start codons attributed automatically by softwares like Glimmer or GeneMark are indicated by a red box and a red circle, respectively. However, both ATG sites lacked correctly positioned ribosome binding site as well as −10 and −35 promoter elements. Most importantly, in both cases, amino acid translation would have resulted in an N-terminus of GNA1870 which lacked the features of a leader peptide, thus preventing the correct prediction of GNA1870 as a lipoprotein. Manual inspection identified the rare GTG start codon in position +1 as the real translation initiation codon. The putative ribosome binding site is shaded and two possible −10 and −35 promoter elements are indicated by dashed lines and dots, respectively.

Although the concept of mining genome information was straightforward, the challenge of whether it could result in the development of a vaccine was not. Indeed, proper cellular localization is the key attribute of a bacterial protein to be considered as a potential vaccine candidate. While proteins located in the cytosol are generally not good immunological targets, surface-associated structures are potentially accessible to the immune system and therefore more likely to induce a functional immune response. Based on this assumption, an *in silico* bioinformatics approach was used to identify novel antigens for vaccine development. The genome was therefore screened systematically to identify proteins predicted to be secreted or exported to the outer membrane, localized in the periplasm or in the inner membrane. Furthermore, selection was also extended to proteins containing amino acid signatures predictive of a possible role in the adhesion to host factors, as well as other virulence mechanisms. This was challenging since the MenB genome consisted of more than 2,000 predicted genes, only a minority of which coded for surface expressed molecules of potential utility as vaccine antigens (33). Although today sophisticated software and dedicated suites of programs exist to accurately predict a protein's cellular localization and potential biological function, this was not the case 20 years ago. In the late 1990s, interrogation of sequence data was in its infancy, the utility of many of the algorithms was not validated and annotations were often misleading. Management and interpretation of about two million base pairs of meningococcal genome sequence data were prone to errors. For instance, prediction of the start codons was based on the identification of the first ATG occurring after a previously identified stop codon. Unfortunately, this did not take into account either the presence of a correctly spaced Shine Dalgarno sequence, or the potential presence of less frequent start codons like TTG or GTG (coding for leucine or valine, respectively). For example, the annotation of GNA1870 (later renamed fHbp) was incorrect as a result of automatic procedures Figure 1 and (35) is now one of the most important meningococcal antigens. The plethora of repetitive DNA elements in the genomes of meningococci was a deterrent to efficient assembly and unambiguous identification of genes because of frame shifts or sequencing errors. In the particular case of MC58 genome annotation, 65 open reading frames that contained stretches of repetitive DNA were identified. Of these, only 16 were previously known; the remainder was discovered through complete genome sequencing (36). The fact that some of the genes with DNA repeats encode for surface associated proteins poses a problem in terms of antigen selection, as phase variable genes, potentially generating escape variants, may not be ideal candidates for vaccine development. Despite these challenges, 18 months after the beginning of sequencing, 600 potential vaccine candidates were identified *in silico*. The highest proportion of these putative candidates was represented by integral membrane proteins (characterized by multiple hydrophobic domains), followed by periplasmic proteins, lipoproteins, and outer membrane and secreted proteins, the latter group representing <15% of the total. Interestingly, only half of the selected gene products displayed homologies to proteins of defined function, whereas the others had no clearly attributable functional role.

**Figure 2:**
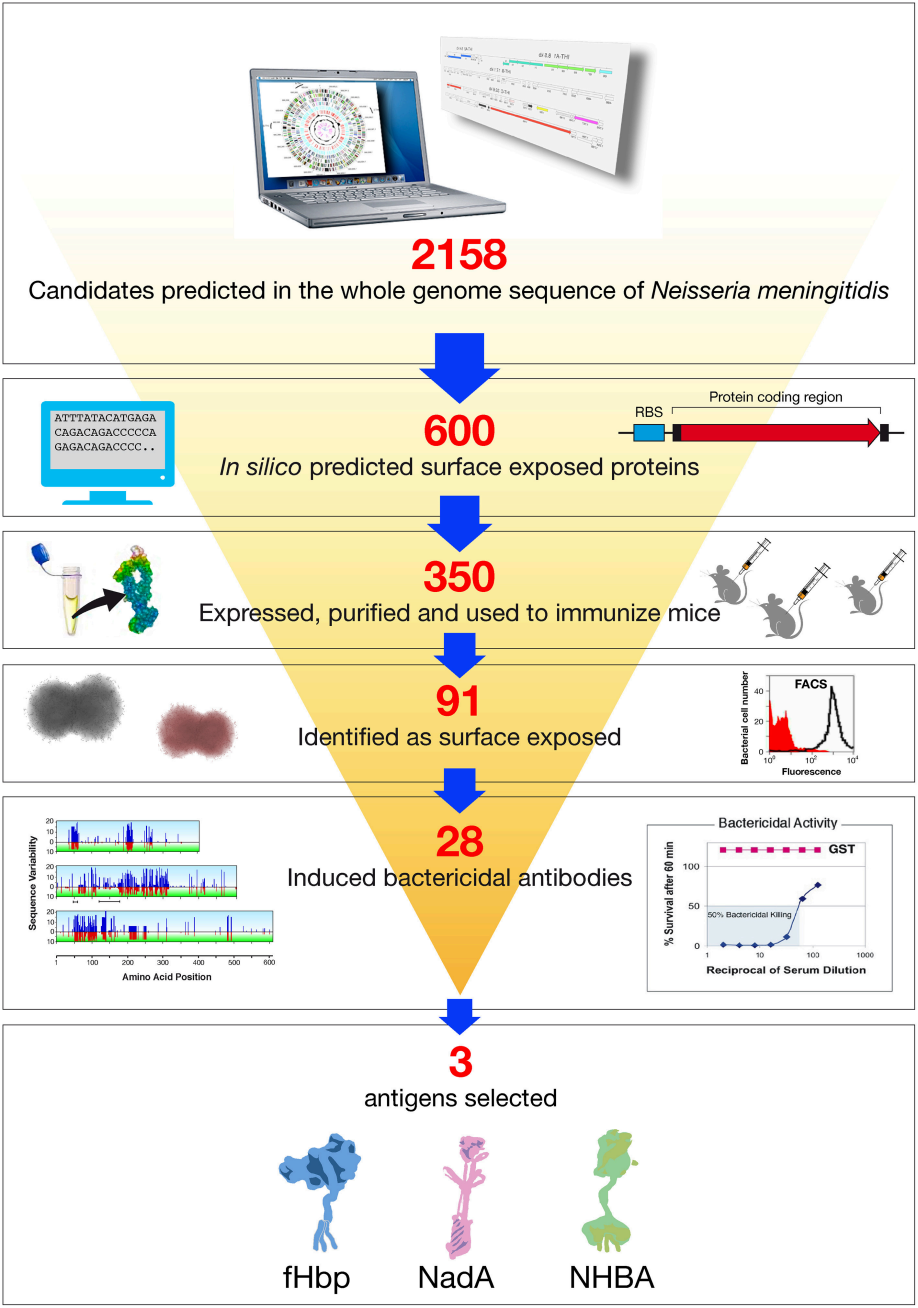
Reverse vaccinology applied to *Neisseria meningitidis* B. Based on the complete genome sequence of *N. meningitidis* strain MC58, 2158 potential open reading frames (ORFs) were identified. *In silico* analysis resulted in the selection of 600 genes potentially encoding for novel surface exposed proteins. These were amplified by PCR and cloned into *Escherichia coli* expression vectors. Three hundred and fifty recombinant proteins were successfully produced, purified, and used to immunize mice. Recombinant protein candidates were then selected based on their surface expression (assessed by FACS), and ability to induce serum bactericidal antibodies (assessed by the SBA serum bactericidal assay) and conservation in a panel of Neisseria strains. The antigens selected by reverse vaccinology were finally prioritized, with NadA, fHbp, and NHBA as the three top antigens.

The genes coding for the 600 potential surface-exposed antigens were amplified from the genome of strain MC58 and cloned in expression vectors to generate Histidine (His) or GST (Glutathione S-transferase) tagged proteins. The fusion protein form, His or GST tagged, which showed higher solubility was purified from *E. coli* and used to immunize mice. The antisera raised against each recombinant antigen were analyzed in a panel of immunological assays: Western blot to confirm that the antigen was expressed in meningococcus and at the predicted molecular weight; flow cytometry by FACS (Fluorescence Activated Cell Sorter) to evaluate accessibility of the antigen to antibody binding on the meningococcal surface, and SBA to assess the ability of the antibodies to bind the antigen and to promote complement mediated bacterial killing. In addition, some of the antigens were tested for their ability to confer protection in the infant rat or mouse septicemia models by active or passive immunizations. Following this approach, 350 of the 600 predicted surface-exposed proteins were successfully expressed in *E. coli* and purified as recombinant proteins. The rate of expression was mainly driven by the intrinsic features of the selected antigens, with those containing more than one predicted transmembrane domain being the most difficult to express. Of the 350 candidate antigens, 91 proved to be surface-exposed, and 28 were able to elicit a bactericidal response. The identification of 28 new bactericidal antigens represented a real breakthrough in the field, considering that in more than 50 years of research only few bactericidal antigens were characterized (32) (Figure 2).

Selecting which of the most promising antigens should be included in the MenB vaccine required an approach informed by sequence analysis of individual surface expressed proteins to assess the extent of their variation within the natural population of meningococcal strains (associated with invasive disease and carriage) and by evaluation of the cross-bactericidal activity of antisera raised against each of the antigens. A collection of strains was assembled by scientists at University of Oxford so as to be representative of the Neisseria species, based on multiple serogroups of *N. meningitidis*. Also included were strains of *Neisseria gonorrhoeae, Neisseria cinerea*, and *Neisseria lactamica* to evaluate the sequence conservation of the top cross-protective antigens. This sequence conservation analysis revealed a substantial degree of variability in at least some of the candidates, suggesting that no single component would be sufficient to induce broad coverage and that a “universal” vaccine should contain multiple antigens (Figure 2).

## Formulating the Multicomponent Vaccine and Functional Characterization of Its Components

**Figure 3:**
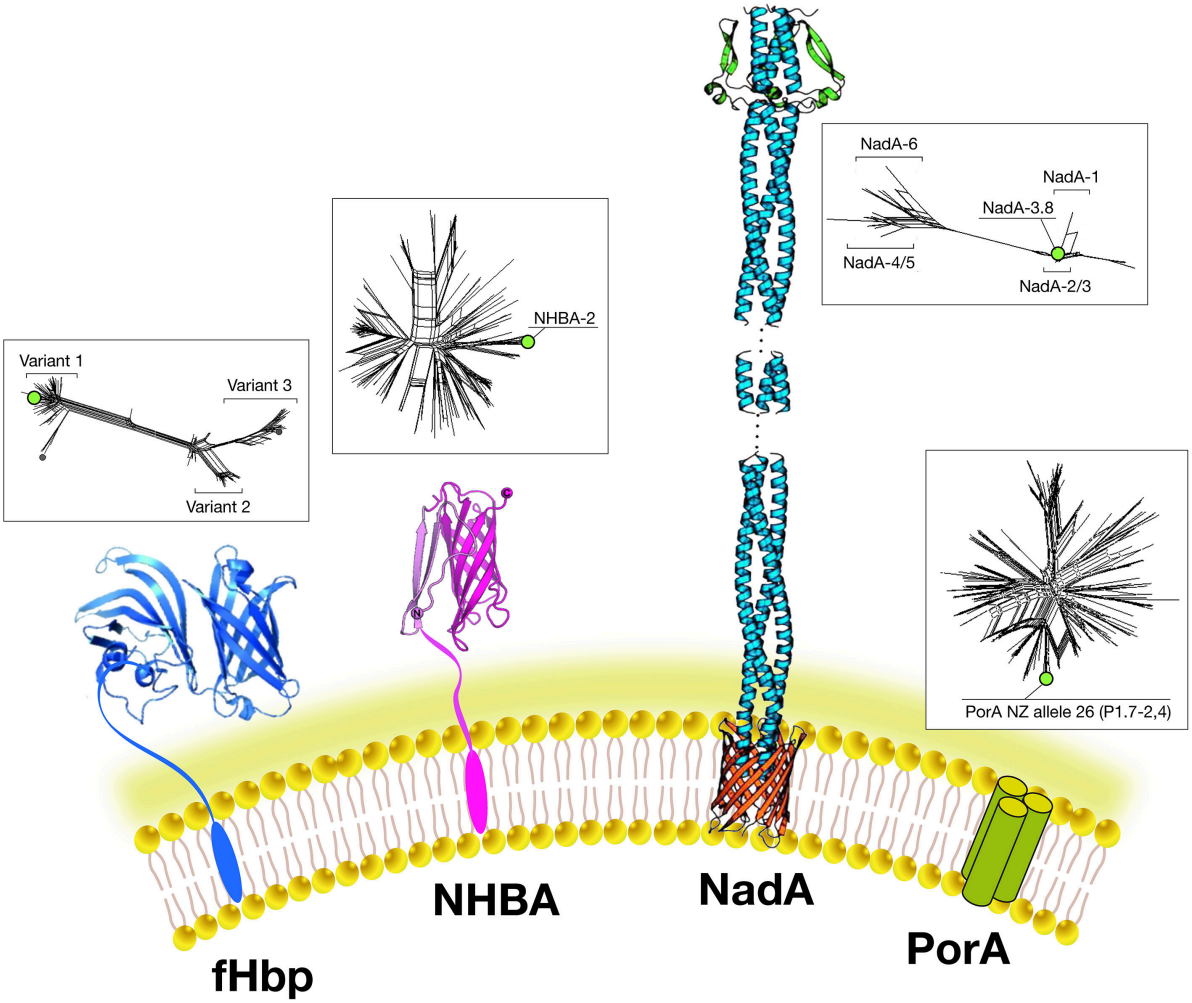
Main components of the 4CMenB vaccine: Schematic representations of the three-dimensional structures of vaccine antigens based on full length x-ray structure of fHbp, depicted in dark blue (37); x-ray structure of the C-terminal domain of NHBA depicted in purple (38, 39). X-ray structures of the trimeric head and stalk domains of NadA molecule shown in light blue; the anchor region (in red) is modeled according to predictions from other outer membrane proteins (40, 41); the N-terminus of the NHBA antigen is predicted as unfolded and has proved resistant to crystallization so far. Schematic model of the trimeric PorA molecule (the immunodominant antigen of the OMV) is depicted in green. Three-dimensional structure of PorA is not available yet. Within boxes are shown phylogenetic trees to indicate the sequence variability of the fHbp, NHBA, NadA, and PorA antigens included in 4CMenB. Green dots indicate the antigen variants present in 4CMenB.

The final choice of antigens to be included in this multivalent vaccine formulation was based on cross-protective ability, assayed by bactericidal activity and maximum coverage of the extensive antigenic variability of meningococcal B (MenB) strains. When initially identified, each candidate antigen was referred to as GNA (Genome derived Neisseria Antigen) followed by a number representing the position of the encoding gene in the genome. The three most promising antigens identified were GNA2132, GNA1870, and GNA1994. In subsequent studies, these three antigens were given names: NHBA, fHbp, and NadA, respectively, based on their functional activity (Figure 3).

NHBA: GNA2132 is a lipoprotein of around 420–490 amino acids and was shown to bind heparin and heparan-sulfate *in vitro*, through an Arginine-rich region. Because of this function, it has been named Neisserial heparin binding antigen (NHBA) (42). Binding to heparin affects survival of Neisseria in a human blood killing assay (42). The Arginine rich region plays also a key role in adhesion to eukaryotic cells (43). The *nhba* gene is ubiquitous in all meningococcal serogroups and is also found in *N. gonorrhoeae* and other commensal Neisseria species. On the basis of the sequence variability, over 400 different peptides have been described and the relationship between sequence variability and cross-protection remains to be defined The NHBA protein has an N-terminal region of approximately 250 residues predicted to be intrinsically disordered, and a highly conserved C-terminal domain (~180 residues), with an 8-stranded anti-parallel β-barrel folding (38, 39). NHBA undergoes proteolytic cleavage by meningococcal NalP protease and by eukaryotic proteases like human lactoferrin, Kallicrein, and the C3 convertase (42, 44).

fHbp: GNA 1870 is a lipoprotein of 253–266 amino acids able to bind human Factor H (FH), an inhibitor of the alternative complement pathway. Because of this activity it has been named factor H binding protein or fHbp (the same antigen discovered using a biochemical approach was named rLP2086) (35, 45). The binding of fHbp to FH enhances meningococcal serum resistance allowing the bacterium to replicate in human blood. The three-dimensional structures of fHbp alone or in complex with domains 6 and 7 of human FH have been solved. Interestingly, the side chains of fHbp that interact with FH resemble the glycosaminoglycan binding region of FH on host cells (37). Therefore, Neisseria is able, through fHbp, to recruit FH by mimicking the host. Sequence diversity analysis allowed identification of three variants, named variants 1, 2, and 3 (or subfamily A and B), serologically distinct and with only a low cross-bactericidal activity between variants 2 and 3 strains. The amount of fHbp expressed by different MenB strains is controlled by the fHbp promoter and can vary of at least 15-fold (46). FHbp contains multiple bactericidal epitopes and bactericidal activity of anti-fHbp antibodies varies according to the genetic diversity and level of expression of fHbp in the different strains (47).

NadA: GNA 1994 is a trimeric autotransporter belonging to the Oca family (oligomeric coiled-coil adhesins) of 323–405 amino acids. It mediates adhesion and invasion to epithelial cells and for this reason it has been named NadA (Neisseria adhesin A) (48). The *nadA* gene is not present in all meningococcal strains, and its presence is mainly associated with the hyperinvasive sequence type 8 (ST-8), ST-11, ST-32, and ST-213 clonal complexes (cc) but is rarely present in ST-41/44 and ST-269 cc isolates (49). Six variants exist of which NadA1, NadA2, and NadA3 are highly immunogenic and induce cross-reactive SBA responses. NadA4 is associated with carriage strains. NadA5 is rare and found in only a few invasive isolates (50). NadA expression levels vary among isolates and expression is upregulated by niche-specific signals via the transcriptional regulator NadR, which binds the NadA promoter and represses transcription. DNA-binding activity of NadR is attenuated by 4-hydroxyphenylacetic acid (4-HPA), a natural molecule released in human saliva, thus leading to the de-repression of *nadA in vivo* (51). Because of this tight regulation, the role of NadA in vaccine coverage may be underestimated *in vitro*. NadA forms stable trimers on the bacterial surface and mediates binding to epithelial cells through interaction with protein receptor molecules differentially expressed by various epithelial cell lines. The three dimensional structure reveals a novel TAA (trimeric auto-transporter adhesins) organization made mostly of a coiled-coil with protruding wing-like structures forming a head-like domain (40).

With the aim of maximizing strain coverage while facilitating large-scale-manufacturing, fHbp, NHBA, and NadA were fused to additional candidate antigens, previously selected based on their ability to induce bactericidal activity and/or protection in animal models. More than 30 protein-protein fusions were generated and analyzed for their biochemical and immunological properties. Based on these analyses, GNA2132-GNA1030 and GNA2091-GNA1870 were the most stable and the most immunogenic in animal testing. Surprisingly, bactericidal activity induced by immunization with fHbp and NBHA was increased when each was fused to other antigens. In contrast, NadA was less immunogenic when fused to other antigens, probably because of the loss of its trimeric structure.

A vaccine consisting of three recombinant proteins, two protein-protein fusions plus a single antigen, named recombinant MenB vaccine (rMenB) was formulated with aluminum hydroxide and used to immunize mice. From a collection of 214 *N. meningitidis* clinical isolates (obtained from Europe, Canada, US, Australia, and New Zealand) to represent the global population diversity of invasive serogroup B isolates, bactericidal assays were performed on 85 strains using rabbit sera as exogenous complement source. The rMenB vaccine induced bactericidal antibodies against 78% of these strains. To improve immunogenicity and potential strain coverage of rMenB, an outer membrane vesicle component obtained from the epidemic New Zealand strain was added to the formulation to create a four component vaccine, called 4CMenB (52).

## From WGS Vaccine Antigen Discovery to a Licensed Vaccine

Each component of the MenB vaccine had to satisfy a plethora of demanding regulatory conditions with respect to safety and immunogenicity, each having complex cost implications with respect to their manufacture and formulation. Six years pre-clinical research on toxicity, stability and immunogenicity were required before the two candidate MenB formulations, rMenB and 4CMenB, were approved for clinical trials that commenced in adults in 2004 (53). Clinical trials of these vaccines in infants began in 2006 (54, 55), bypassing the conventional pathway involving a step-wise decrease in the age of subjects; primary school to pre-school to toddler to infant. This accelerated program in part reflected an awareness from the experience with OMV based vaccines that the breadth of immune response to meningococcal antigens was likely to be age dependent. Therefore, there was an imperative to evaluate the breadth of the immune response (i.e., cross-reactivity with non-vaccine variants of the vaccine antigens) in the age group most at risk of disease early in the vaccine's clinical testing.

A major challenge for these clinical trials was how to determine the immunogenicity of the vaccine candidates. In contrast to the previously licensed meningococcal glycoconjugate vaccines for which target antigens (distinct polysaccharide capsules) were invariant structures, the outer membrane proteins contained in rMenB and 4CMenB are variable in both primary sequence and level of expression. As in pre-clinical studies, this required judicious selection of MenB isolates on which to perform bactericidal assays, such that these distinct strains were representative of the diversity of invasive disease MenB target antigens. A further constraint was the small amount of serum that could be obtained in clinical trials involving infants; this limited the number of assays that could be performed to evaluate immunogenicity.

Thus, while the initial phase 1 human studies tested the immune response against 15 strains, the early phase 2 infant studies tested post-immunization sera against a panel of 7 strains (54, 55). Four of these strains were reference strains chosen to demonstrate the immunogenicity of individual vaccine antigens (fHbp, NHBA, NadA, and PorA) (Table 1). Immunization with 4CMenB induced bactericidal antibodies against a greater proportion of meningococcal strains than did rMenB. These findings were the basis of the decision to select 4CMenB, rather than rMenB, for further clinical development. A series of clinical trials evaluated how the immune response to the 4CMenB vaccine antigens was influenced by factors such as age of administration, concomitant vaccine use and lot-to-lot consistency.

**Table 1:** MenB indicator strains used to assess bactericidal activity induced by immunization with 4CMenB.

**Classification of serogroup B meningococcal strains**			**Antigen gene sequence variant**			
**Designation**	**Sequence type**	**Clonal complex**	**PorA (VR2)**	**fHBP**	**NadA**	**NHBA**
44/76-SL	32	32	P1.16	1.1	–	3
5/99	1349	8	P1.2	2.23	2	20
NZ98/254	42	41/44	P1.4	1.14	–	2
M10713	136	41/44	P1.16-3	2.24	–	10

*H44/76 is the indicator strain for fHbp, 5/99 for NadA, NZ98/254 for PorA and M10713 for NHBA*.

4CMenB was approved in Europe in 2013 and introduced in the National Immunization Program in the UK starting from September 2015; the vaccine was offered to all newborns using a 2, 4, and 12 months schedule. The effectiveness against IMD measured at eleven months after the study and five months after the second vaccination, was 82.9% (95% CI: 24.1 – 95.2). The wide confidence limits reflect the challenges of interpreting the post-implementation data in the short term, given the relatively small numbers of cases and the temporal fluctuations in rates of disease that are typical of meningococcal disease epidemiology. Nonetheless, following implementation of 4CMenB vaccine, the number of cases in vaccine-eligible infants was reduced by 50% (95% CI 36–71; *p* = 0.0001), compared to the pre-vaccine period. The long term impact of 4CMenB vaccine implementation on disease burden, disease severity and safety will continue as part of the National Surveillance program (56). There were also extensive phase 2 and 3 studies to investigate the safety and tolerability of the vaccine, of particular importance given the previous experience of the reactogenicity of OMV vaccines. These clinical trials, involving approximately 7,400 children under 11 years of age prior to licensure in Europe “EMEA assessment report November 2012. http://www.ema.europa.eu/docs/en__GB/document__library/EPAR__-__Public__assessment__report/human/002333/WC500137883.pdf”), demonstrated that ~60% of children receiving 4CMenB concomitantly with DTaP (Diphteria, Tenanus, and acellular Pertussis) and pneumococcal conjugate vaccines experienced fever, compared to ~30% when these vaccines were given without 4CMenB. In infants, local and systemic reactions appeared to be more frequent when 4CMenB was co-administered with other vaccines, but medical attendance after vaccination and fever-related serious adverse events (SAEs) were rare. The occurrence of febrile seizures was comparable to that reported from other combination vaccine studies. Two cases occurred within 24 h after the first and another two cases after the second vaccination with 4CMenB and routine vaccines. These cases were assessed as possibly associated with vaccination but were deemed as mild and resolved spontaneously. Most other adverse events were common childhood illnesses or events consistent with solicited reactions and resolved at final follow up (57). After its introduction in the routine UK immunization program, there was an increase in presentations to Accident and Emergency and in hospital admissions for transient adverse events following immunization (58). In contrast, a suggestion of an association with Kawasaki disease in early clinical trials was not supported by post-implementation surveillance (59).

To obtain licensure by the European Medicines Agency and multiple other regulatory agencies internationally, a major challenge was how to estimate the protective potential of 4CMenB against invasive disease. Owing to the low incidence of meningococcal disease, classical efficacy studies were impractical. Thus, SBA using human complement was used to estimate vaccine functional immunity against invasive meningococcal disease. But, because the MenB strains that cause invasive meningococcal diseases are highly diverse with respect to the quantity and immunological cross-reactivity of the vaccine antigens expressed, estimating the effectiveness of the vaccine required performing SBA against large numbers of isolates, an undertaking that was judged to be impractical. Therefore, an innovative method was developed to assess coverage and predict effectiveness of the 4CMenB vaccine. This assay, called MATS (Meningococcal Antigen Typing System), correlated information on the quantity and quality of the antigens expressed by individual MenB strains and the potency of the immune response elicited by the vaccine based on bactericidal assays.

**Figure 4:**
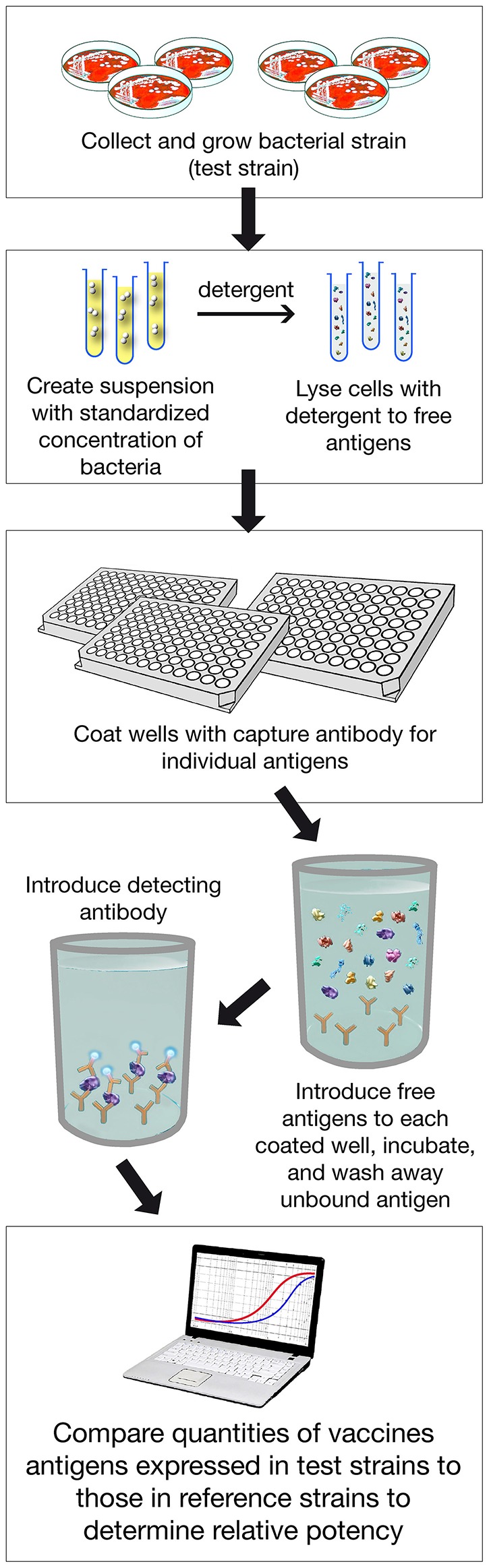
Assay Procedure overview of MATS ELISA. Various steps of the assay are shown. In addition PorA genotype is defined with classical pCR.

**Figure 5:**
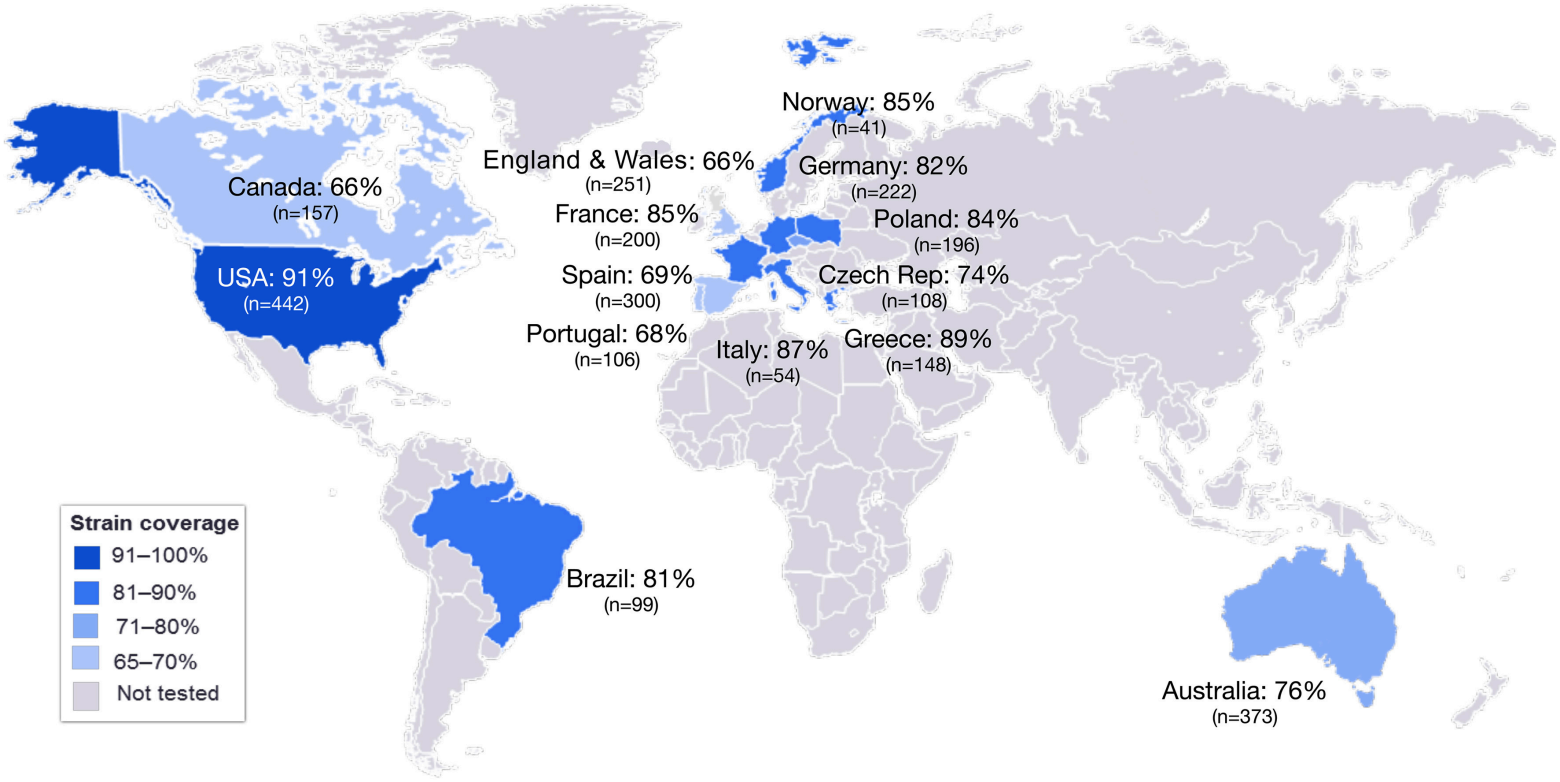
MATS coverage estimates by geographic region. This map schematically represents the results of MATS coverage estimates generated on MenB isolates from different countries colored in blue and scaled according to coverage estimates. Countries for which MATS estimates are not available are colored in gray. In each box is shown the MATS prediction (%) of strain coverage for each country, along with the number (*n*) of strains tested.

MATS is based on the assumption that a given MenB strain is susceptible to killing by vaccine-induced antibodies, providing that this strain expresses one or more surface proteins in sufficient amounts so as to be adequately cross-reactive with a vaccine component (Figure 4). To develop the MATS assay, ELISA reactivity with antisera raised against fHbp, NHBA, and NadA expressed by each tested strain was compared to antigen specific reference MenB strains, a metric called relative potency (RP). Coverage of each individual strain is assumed if the RP is higher than an antigen-specific positive bactericidal threshold (PBT), defined for each antigen on the basis of bactericidal activity of infant sera against a panel of 57 serogroup B strains. PorA cross-reactivity is evaluated by exact sequence matching to PorA P1.4 vaccine serosubtype (60, 61). The MATS assay has been transferred to national reference laboratories in Europe, US and Australia. Worldwide, MATS-predicted coverage afforded by 4CMenB has been estimated at 66% in Canada (62) 68% in Portugal (63), 69% in Spain (64), 74% in Czech Republic (65), 76% in Australia (66), 78% in other 5 European countries (60), 81% in Brazil (66), 84% in Poland (67), 89% in Greece (68), and 91% in US (69). Finally, in England, Wales and Northern Ireland, the MATS estimate of coverage was measured at 73 and 67%, respectively, on different strain collections of 2007–2008 and 2014–2015 the latter representing the baseline before vaccine implementation (56) (Figure 5). Indeed, several publications (70, 71) claim a potential underestimation of MATS predicted coverage estimates, due to a series of reasons: (i) MATS provides an estimation of the contribution of each antigen independently, therefore the synergistic effect of antibodies recognizing different antigens is not measured; (ii) the NadA-mediated contribution to protection is underestimated as NadA expression is downregulated in the *in vitro* conditions in which MATS is performed, compared to expression of the antigen *in vivo* (51); (iii) the contribution of OMV to protection is limited to the presence of a matched PorA antigen, although it is commonly accepted that PorA-independent protection can be afforded by OMV against some strains.

The underestimation of protection predicted by MATS was further supported by a study performed in the UK on a panel of circulating clinical strains where the MATS predicted coverage was 73%, while the SBA showed 88% strain coverage (70). Similar data were also generated on a panel of MenB strains from Spain, showing that isolates found negative in MATS were in fact killed by sera of adolescents and infants immunized with 4CMenB (64). The overall underestimation has more recently been confirmed by the preliminary “real-world” effectiveness of 82.9% based on the results of the routine infant immunization with 4CMenB in the UK (56).

In Canada, 4CMenB was licensed in 2013 for use in 2 months to 18 years old. A mass vaccination campaign, targeting individuals aged 2 months to 20 years was implemented in the Saguenay-Lac-Saint-Jean region of Québec in 2014 to control the high incidence rate of MenB disease. Following the campaign, the incidence in the region decreased, with no cases reported in the vaccinated individuals but with two cases occurring among the unvaccinated (72). In US, 4CMenB has been authorized in 2015 and recommended for use in the 10–25 years old as two doses vaccine. It has been used to control MenB outbreaks at University and college campus in Oregon, New Jersey and California (73, 74). No cases of MenB disease have been reported so far in vaccinated individuals, suggesting that the vaccine is effective in this age group. Moreover, when the immune responses induced by 4CMenB during the outbreak in Princeton was measured, 33% of 4CMenB vaccines showed no SBA against the outbreak strain, although no cases of meningococcal disease caused by N. meningitidis B were reported among vaccinated student (73).

## Discussion

The licensure in 2013 of the four component MenB vaccine (Bexsero) was the culmination of a scientific collaboration between university and industry-based scientists. The former provided cutting edge genomic, genetic, and clinical trials expertise; the latter undertook the vital high-throughput, “brute force” evaluation of hundreds of candidate antigens discovered through genomics, the in-depth characterization of the functional and immunological properties of the selected vaccine antigens and then stage-managed the pre-clinical and clinical testing required to obtain licensure. The facilitating technological breakthrough of WGS of bacterial pathogens came about through a former NIH academic, Craig Venter, who used his entrepreneurial vision to set up TIGR, the sequencing facility that made the MenB project possible. The 2018 Gairdner Award to Rino Rappuoli https://www.aditecproject.eu/2017/05/04 who oversaw this academic-commercial partnership was fitting recognition of his role in driving through the innovative application of genomics to antigen discovery, the first example of what has become known as “reverse vaccinology.”

4CMenB represents a striking departure from the successful research and development platform that resulted in several, highly safe and effective conjugate meningococcal vaccines (against meningococcal serogroups A, C, W, and Y strains) formulated through covalent chemical linkage of different serogroup capsular polysaccharides to proteins. Although each of the meningococcal capsular polysaccharides shows strikingly distinct chemical compositions, each is an invariant structure whose target epitopes do not change over time or region. Diversity in the “carrier” proteins used to formulate conjugate vaccines are not problematic providing that these variations do not interfere with their role in recruiting T-cell help. But for vaccines, such as 4CMenB, where the antigens inducing protective immunity are proteins, the scenario is fundamentally different. The amino acid sequence of each of the protein antigens is highly variable, a consequence of their location on the bacterial surface where exposure to immune responses drives selection and fixation of diversity in circulating strains of meningococci. The multivalent protein vaccine, 4CMenB, is not without precedent; the several acellular pertussis vaccines have formulations consisting of up to 5 proteins, although in retrospect there was inadequate appreciation of the complications of allelic variation of these vaccine proteins. Loss or gain of DNA has over many years impacted on the effectiveness of *B. pertussis* vaccines (75), but its population structure is clonal (76), so there is no recombination and the rate at which antigenic variation accumulates is very gradual over time. In contrast, genetic variation in meningococci occurs predominantly through recombination, not intra-genomic mutations. Thus, within the natural population of meningococci, there is frequent horizontal transfer of DNA, mainly through DNA transformation, not only between distinct genotypes of *N. meningitidis*, but also from other sub-species of Neisseria and, rarely, other distinct bacterial species. For example, conserved homologs of the *nhba* gene have been found in commensal Neisseria species, such as *N. lactamica, N. polysaccharea*, and *N. flavescens* (77). This finding is relevant because of the potential “selective impact” that a NHBA-containing vaccine could have not only on encapsulated meningococcal strains, which are potentially pathogenic, but also on the commensal flora. This rampant recombination has major implications in that to be an effective vaccine, 4CMenB must elicit antibodies that protect against an enormous diversity of circulating meningococcal strains in a microbial population that is also constantly evolving over time. There was a requirement to develop a vaccine typing scheme to characterize any carriage or invasive meningococcal isolate. This effort resulted in the identification of the MATS assay as predictor of vaccine coverage. Since MATS can be applied only to cultivable strains, and considering that more than 50% of cases do not have an isolate, genomic driven predictor of coverage, such as BAST [Bexsero Antigen Sequence Typing (78)] or gMATS (genetic MATS), under development, will be instrumental to more precisely evaluate vaccine coverage.

The need for new lines of thinking emerged early in the pre-clinical phases of 4CMenB development. Given the rarity of IMD [0.5–1 case per 100,000 per annum in Europe and the Americas] (79), reliance on a surrogate of protection to select appropriate protein antigens was paramount. The acceptance of SBA as a gold-standard surrogate of protection against meningococcal surface proteins (80) by scientists and regulatory authorities was a major milestone. It meant that the pre-clinical and clinical studies could proceed to licensure without the need for the conventional phase 3 efficacy trials for which cases of invasive disease provide the key metric. Given the logistics, expense, and large numbers of subjects required to assess efficacy, it was considered unlikely that any such clinical trial could be carried out.

Indeed, overall, the complexity of 4CMenB and the pathway to licensure made unprecedented demands on both the vaccine development teams and the regulatory authorities. Dialogue and an iterative scientific interchange was essential to address all regulatory requirements and translate them into practice.

The pathway of reliance on phase 2 immunogenicity studies of 4CMenB, backed by SBA and the derivative innovation of the MATS assay, was enormously facilitated by the previous experience with the MenC conjugate vaccines whose successful implementation in the routine UK immunization programme was a game changer (81). The way forward for 4CMenB has followed along similar lines, but has been immensely more complicated. For the reasons discussed above, estimates of effectiveness for the invariant meningococcal C polysaccharide vaccine antigen were far simpler than for the variable four protein antigens of 4CMenB. One major lesson emanating from experience with the conjugate vaccines in general, specifically exemplified by data on serogroup C meningococcal conjugates, has been the extent to which their success depends on indirect, or herd, immunity (82). The mechanism of indirect protection is through curtailing transmission of meningococci and therefore decreasing the probability of new acquisitions and the risk of invasive disease. A UK study estimating the effect of meningococcal vaccines on herd protection against *N. meningitidis* in University students, showed that both, 4CMenB and MenACWY vaccines induced carriage reduction only for a subset of *Neisseria* strains, 4–12 months after vaccination (83). To date, the impact of 4CMenB on carriage of meningococci remains uncertain. Further studies are required, including those that ascertain whether there is an impact of the vaccine on bacterial load.

The inclusion of 4CMenB in the UK routine infant immunization programme since October 2014 allows post-implementation surveillance that over many years will provide crucial information on its effectiveness and duration of protection. In being given routinely only to infants, 4CMenB is not expected to prevent cases of meningococcal IMD in older children, adolescents or adults. In addition, estimates of protection, based on WGS, hSBA, and MATS, indicate that a proportion of strains lack a biologically relevant match to the antigens in the vaccine. As an example, the proportion of strains negative in hSBA in an UK strain panel was 12% (70). These *in silico* and *in vitro* predictions of vaccine effectiveness must be interpreted with caution since these metrics for estimating protection have not been validated. Two fundamentals of determining vaccine effectiveness are accurate information on immunization uptake and a robust system for disease notification. These data enable the calculation of vaccine effectiveness, as the likelihood of a child with disease being immunized (i.e., a vaccine failure) or unimmunized can be compared to that in the general population. This so-called screening method (84) was used to establish the effectiveness of the UK MenC conjugate vaccines and the OMV Vaccine in New Zealand. It is crucial to have consensus on the definitions of what constitutes a case of meningococcal disease and an “immunized” child. To this end, organizations such as the European Centre for Disease Prevention and Control (ECDC) have provided definitions (85). More problematic is the definition of vaccine failure, which can be considered at either an individual or population level. At an individual level, not every case of MenB disease in an immunized child should be seen as a vaccine failure as the disease causing strain may not have expressed the vaccine target antigens. Thus, defining what criteria should be used to identify vaccine failures remains an exercise in pragmatism, dependent for validation on the accumulation of real-time data on rates of meningococcal disease, information that will require many years of surveillance using the screening method. Further complications include the changing incidence of IMD in the UK, a sharp decline in recent years (86) and the fact that in about half of all cases, no organism is isolated and confirmation is based on PCR (87), presenting major challenges to complete characterization of the target antigens of infecting meningococcal genotypes, in terms of expression and surface accessibility. 4CMenB is an exemplar of what can be truly considered a new era in vaccines.

## Author Contributions

All authors listed have made a substantial, direct and intellectual contribution to the work, and approved it for publication.

### Conflict of Interest Statement

VM and MP are employees of the GSK group of companies. EM has a consultancy contract with GSK.
